# Fluctuation-based outlier detection

**DOI:** 10.1038/s41598-023-29549-1

**Published:** 2023-02-10

**Authors:** Xusheng Du, Enguang Zuo, Zheng Chu, Zhenzhen He, Jiong Yu

**Affiliations:** School of Information Science and Engineering, Xinjiang University, Ürümqi, 830046 China

**Keywords:** Electrical and electronic engineering, Mechanical engineering

## Abstract

Outlier detection is an important topic in machine learning and has been used in a wide range of applications. Outliers are objects that are few in number and deviate from the majority of objects. As a result of these two properties, we show that outliers are susceptible to a mechanism called *fluctuation*. This article proposes a method called fluctuation-based outlier detection (FBOD) that achieves a low linear time complexity and detects outliers purely based on the concept of fluctuation without employing any distance, density or isolation measure. Fundamentally different from all existing methods. FBOD first converts the Euclidean structure datasets into graphs by using random links, then propagates the feature value according to the connection of the graph. Finally, by comparing the difference between the fluctuation of an object and its neighbors, FBOD determines the object with a larger difference as an outlier. The results of experiments comparing FBOD with eight state-of-the-art algorithms on eight real-worlds tabular datasets and three video datasets show that FBOD outperforms its competitors in the majority of cases and that FBOD has only 5% of the execution time of the fastest algorithm. The experiment codes are available at: https://github.com/FluctuationOD/Fluctuation-based-Outlier-Detection.

## Introduction

The outlier detection problem can be defined as follows: given a dataset ***X*,** find objects that are considerably dissimilar, exceptional and inconsistent with respect to the remaining majority of objects^1^. Detecting outliers is important for many applications, such as financial fraud detection^2,3^, network analysis^4,5^, medical diagnosis^6,7^, intelligence agriculture^8,9^ and even the discovery of new stars in astronomy^10,11^.

The efficiency and effectiveness of most existing outlier detection methods, including distance-based and density-based methods, may be severely affected by increasing data volumes and dimensions due to the “curse of dimensionality”. Clustering-based, classification-based, and autoencoder-based algorithms are usually byproducts of algorithms originally designed for purposes other than outlier detection. This leads to these methods often underperforming and detecting too few outliers. For isolation-based methods, when the number of objects is too large, normal objects interfere with the process of isolation and reduce the ability to detect outliers. To address the above problems, we propose a fluctuation-based outlier detection algorithm. Our motivation is to customized design an outlier detection algorithm with low time complexity and independent of the curse of dimensionality.

Outliers have two distinct properties: (1) they represent a very small proportion of the overall dataset; (2) the feature values deviate significantly from the majority objects. If the neighbors of an outlier are overwhelmingly normal objects, the fluctuations, or degree of change in its feature values when the feature values of its neighbors are aggregated, will be more different from the fluctuations of its neighbors. Normal objects, which come from similar generative mechanisms, will have higher similarity between their feature values. When a normal object aggregates the feature values of its neighbors (the majority of which are normal objects), its fluctuations will be less different from those of its neighbors overall. Although fluctuation-based outlier detection is a very simple method, we show in this article that it is both effective and efficient in detecting outliers.

We summarize the main contributions of this work as follows:

1. We first proposed a concept of fluctuation. The changes that an object causes in itself by aggregating the feature values of its neighbors are called fluctuations. The more similar the fluctuation values of an object and its neighbors are, the less likely it is to be an outlier.
2. Our proposed FBOD algorithm does not need to calculate the distance or similarity between objects, which reduces the computational cost and improves the detection efficiency.
3. FBOD has a linear time complexity with a small constant, which means FBOD has the capacity to scale up to handle large data sizes and extremely high-dimensional problems.
4. We performed extensive experiments on eight real-world datasets and three video datasets to demonstrate the effectiveness of our method. The experimental results demonstrated the superiority of the proposed algorithm over several state-of-the-art algorithms.

## Related work

The earliest studies on outlier detection date back to the 1960s, when researchers considered outliers to be noise and not to contain any valuable information. However, “one person's noise may be another person's signal”^12^, in-depth analysis of outliers can reveal information of significant value hidden in the data^13^.

Many well-established outlier detection algorithms have been devised, and they come in a wide variety with varying performance. Based on their core detection principles, we have divided them into two categories, which are: (a) classical outlier detection; (b) deep learning-based outlier detection. Classical outlier detection methods are those traditional algorithms that do not use deep neural networks. It is worth noting that some deep learning based outlier detection methods are used in combination with classical outlier detection methods.

### Classical outlier detection

Since classical outlier detection algorithms have a large research history, we subdivide them into seven categories, which are: Statistical-based; Distance-based; Graph-based; Density-based; Clustering-based; Ensemble-based; Isolation-based.

*Statistical-based* In statistical-based outlier detection methods, the objects are sometimes modeled using a Gaussian distribution or regression methods, some objects can be labeled as outliers depending on the fitting degree with the distribution model. The disadvantage of statistical-based outlier detection is when faced with high-dimensional datasets, the processing time is sharply elevated^14^.

*Distance-based* The core idea of the distance-based approach is derived from the definition of outlier points by Knorr et al. Knorr and Ng define “a point *x* in a dataset ***X*** is a *DB*(*g*, *D*) outlier if at least a fraction g of the points in ***X*** lies at a greater distance than *D* from *x*”^15^. These methods are difficult to handle large scale and complex data because of their high consumption of computational resources and their inability to implement batch computation^16^.

*Graph-based* Cut-point clustering (CutPC) algorithm can automatically perform outlier detection without any user-set parameters. This method can identify clusters with arbitrary shapes and detect outliers. However, CutPC is not effective in detecting datasets with large density variations and is also affected by the curse of dimensionality^17^.

*Density-based* This type of approach usually relies on the assumption that “outliers are in sparse regions of the data space, far away from highly dense clusters of normal objects.” Typical representative algorithms are Local Outlier Factor (LOF)^18^, Connective Outlier Factor (COF)^19^, and Local Distance-based outlier Factor (LDOF)^20^. The density-based approach has the same shortcomings as the distance-based approach, i.e., high computational resource requirements and difficulty in handling complex high-dimensional data.

*Clustering-based* Clustering-based outlier detection approaches usually take a two-step approach: grouping the data with clustering and analyzing the degree of deviation based on the clustering results. The representative algorithms are K-means^21^, Ordering Points to Identify the Clustering Structure (OPTICS)^22^, etc. Their performance is highly dependent on the effectiveness of capturing the cluster structure of normal objects. In clustering-based methods, outliers are binary. There is no quantitative indication of the object’s outlierness^23^.

*Ensemble-based* The outlier detection algorithm for ensemble learning works by focusing on combining the outputs of different types of algorithms to build stable integrated models for efficient detection of detecting outliers. They are more stable and give better predictive models, but for real-world datasets, outlier analysis can be very complex to evaluate, and selecting the right meta-detectors is a difficult task^24^.

*Isolation-based* Isolated forest is widely used in industry. Isolation-based method determines those objects that are more easily to be isolated as outliers by constructing isolated trees and ensemble them into an isolated forest. However, the IForest algorithm is difficult to detect outliers that are mixed around normal objects^25^.

### Deep learning-based outlier detection

In various practical applications, deep outlier detection offers significantly better performance than traditional outlier detection in solving challenging detection problems. Deep outlier detection includes three conceptual paradigms: (1) deep learning for feature extraction, (2) normality learning for feature representation and (3) end-to-end outlier scoring learning.

Deep learning achieves state-of-the-art performance for outlier detection in structured, high-dimensional data. In most deep learning-based approaches, autoencoders play a central role, usually assuming that outliers are difficult to reconstruct by the autoencoder^26–28^. The autoencoder method does not restrict data types, but relies on data patterns, which can cause large deviations if uncommon data patterns are encountered. In recent years, some researchers have used generative adversarial networks (GAN) for outlier detection^29^. The representative one is the Single-objective generative adversarial active learning (SO-GAAL) algorithm^30^, which can generate a large number of potential outliers, to balance the outlier detection problem into a binary classification problem. However, GANs are difficult to train, and in practice, multiple generators need to be trained to generate higher quality potential samples, which results in lower detection efficiency of the algorithm.

At the same time, many researchers have started to apply graph neural networks^31^ to various types of domains due to their demonstrated power in processing graph data. In the field of outlier detection, most researchers have focused on the use of graph neural networks for outlier detection in graph data^32–34^. However, there is a lack of research related to the application of graph neural networks to outlier detection in tabular datasets.

## Model

**Figure 1:**
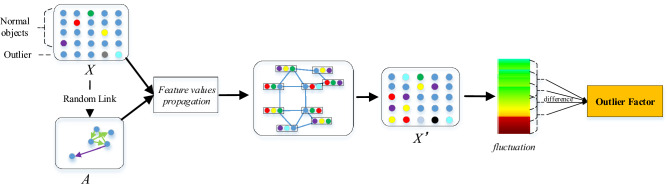
Illustration of the fluctuation-based outlier detection mechanism. FBOD compares the difference between the fluctuation of an object and its neighbors and determines the object with a larger difference as an outlier.

Let ***X*** = {***x***_**1**_**, *x***_**2**_**, *x***_**3**_**…, *x***_***n***_} be the given dataset of a *D*-variate distribution. The core idea of fluctuation-based outlier detection is as follows. The fluctuations produced by normal objects aggregating the feature values of their neighbors on themselves are similar to the fluctuations of their neighbors (the neighbors are basically normal objects). The fluctuations produced by outliers aggregating the feature values of their neighbors on themselves are completely different from the fluctuations of their neighbors. The fluctuation-based method compares the difference between the fluctuation of an object and its neighbors and determines the object with a larger difference as an outlier. Figure 1 shows the overall architecture of the FBOD algorithm.

As shown in Fig. 1, the FBOD algorithm first constructs the connection relationship between objects by sampling (graph generation); then propagates the feature values based on the graph, each object aggregates the feature values of its connected nodes and affects itself; then calculates the fluctuation value of the object; and finally defines the outlier factor of the object based on the difference between the fluctuation value of the object and its neighbors. The higher the outlier factor, the more likely the object is an outlier.

The fluctuation-based method consists of three parts: (a) graph generation; (b) feature value propagation; and (c) outlier factor. We will describe these three parts in detail below.

### Graph generation

**Figure 2:**
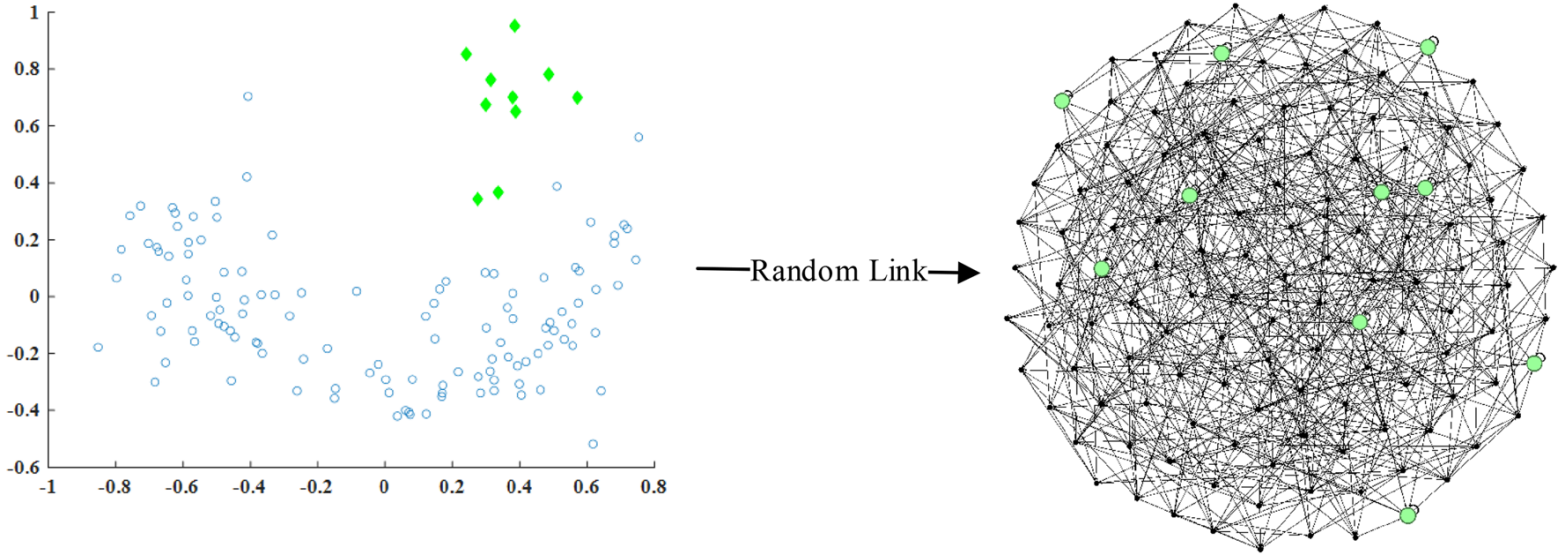
Illustration of the graph generation mechanism. The graph generated from real-world dataset wine (*k* = 5); the solid green circles indicate the outliers. On the right side of this figure, each object has 5 indegrees and is connected to itself.

In the Euclidean structure dataset, objects are independent of each other, and there is no connection relationship. We propose a random link method to convert the original unconnected relationship into a connected relationship, called graph generation. The graph generation mechanism is represented in Fig. 2.

#### Definition 1

*k* neighborhood of an object ***x***_***i***_.

Randomly select *k* objects from the dataset ***X***; the set formed by these *k* objects is named the *k* neighborhood of an object ***x***_***i***_ and denoted as *N*_*k*_(*x*_*i*_) ($x_{i} \notin N_{k} \left( {x_{i} } \right)$ and ${|}N_{k} \left( {x_{i} } \right){| = }k$). The process of constructing its *k*-neighborhood for any object is equivalent to subsampling the dataset ***X*** once.

**Figure 3:**
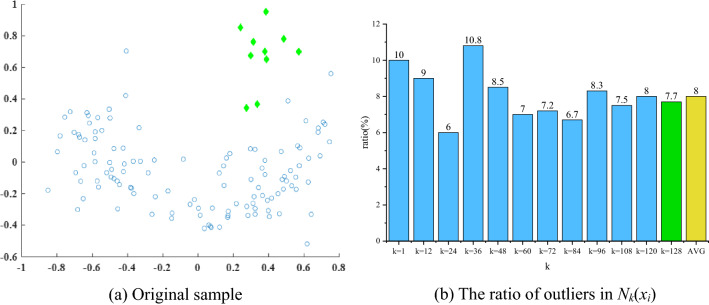
Using the real-world dataset wine to describe the ratio of outliers in *N*_*k*_(*x*_*i*_) is similar to the original ratio. (**a**) Includes 129 objects with 10 outliers, and the rate of outliers is 7.75%. (**b**) Shows that by randomly selecting *k* objects from (**a**) and constructing the neighborhood of *x*_*i*_, the average rate of outliers in *N*_*k*_(*x*_*i*_) are 8% (when *k* takes different values, the average value is taken of 10 executions).

Based on the fact that there is very few outliers in the dataset, even with the random link method, it is still possible to ensure that the *k*-neighborhoods of each object will contain mostly normal objects. Figure 3 describes the ratio of outliers in *N*_*k*_(*x*_*i*_) is similar to the original ratio.

**Figure 4:**
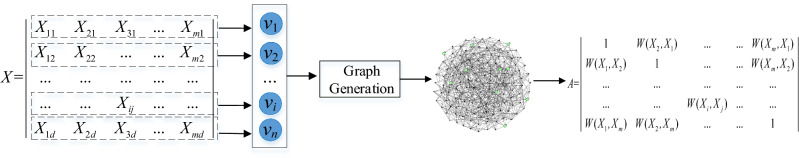
Graph generation.


Graph generation consists of three main steps: (1) construct *N*_*k*_(*x*_*i*_); (2) construct a directed edge from the object's neighbors to itself; (3) store the constructed graph in the adjacency matrix ***A***. The flow chart of graph construction is shown in Fig. 4.

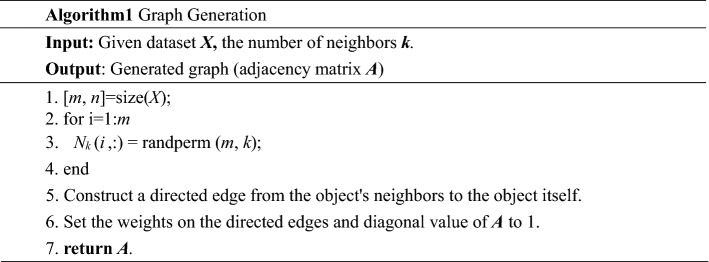


### Feature value propagation and fluctuation

Let the graph generation from ***X*** be ***A***. Design the feature value propagation as:

1 $$X^{\prime} = X*A$$

**Figure 5:**
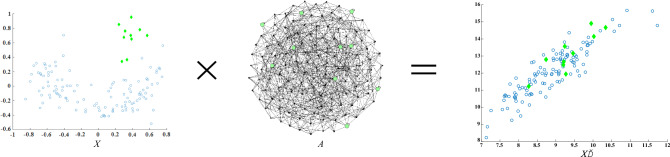
An illustrate graph of feature values propagation. The rightmost side of Fig. 4 shows the distribution of ***X*** after feature value propagation. When the object aggregates the feature values of other objects, its own feature values change, resulting in a significant change in the overall data distribution.

We provide an example to illustrate feature value propagation in more detail. Let $$X = \left[ \begin{gathered} x_{11} \hfill \\ x_{12} \hfill \\ \end{gathered} \right. \, \left. \begin{gathered} x_{21} \hfill \\ x_{22} \hfill \\ \end{gathered} \right]$$. Each column in ***X*** represents an object, and each object has two features. $$A = \left[ \begin{gathered} 1 \hfill \\ W(x_{1} ,x_{2} ) \hfill \\ \end{gathered} \right. \, \left. \begin{gathered} W(x_{2} ,x_{1} ) \hfill \\ 1 \hfill \\ \end{gathered} \right]$$, $$X*A = \left[ \begin{gathered} x_{11} *1 + x_{21} *W(x_{1} ,x_{2} ) \hfill \\ x_{12} *1 + x_{22} *W(x_{1} ,x_{2} ) \hfill \\ \end{gathered} \right. \, \left. \begin{gathered} x_{11} *W(x_{2} ,x_{1} ) + x_{21} *1 \hfill \\ x_{12} *W(x_{2} ,x_{1} ) + x_{22} *1 \hfill \\ \end{gathered} \right]$$. After feature value propagation, object *x* contains not only its own feature value but also the feature value passed by its neighbors. We give an example to illustrate the feature values propagation in Fig. 5.

#### Definition 2

Fluctuation.

Let *x*_*i*_*'* denote *x*_*i*_ after feature value propagation; then:

2 $$fluctuation(x_{i} ) = \sum\limits_{d = 1}^{D} {\frac{{x_{id} }}{{x_{id}^{\prime } }}} { = }\sum\limits_{d = 1}^{D} {\frac{{x_{id} }}{{x_{id} { + }\sum\limits_{k = 1}^{k} {N_{k} (x_{i} )_{d} } }}}$$

In Eq. (2), $N_{k} (x_{i} )_{d}$ denotes the feature value of the *k*th neighbor of object *x*_*i*_ in the *d*th dimension. From section "Graph generation", it is clear that the majority of those contained in *N*_*k*_(*x*_*i*_) are normal objects. Since the feature values of normal objects are similar and the feature values of outlier and normal objects have large differences, we can deduce that:

3 $$\begin{array}{*{20}l} {\left| {\sum\limits_{k = 1}^{k} {N_{k} (x_{i} )_{d} } - k*x_{id} } \right| \approx {0}} \hfill & {if} \hfill & {x_{i} \in normal \;object} \hfill \\ {\left| {\sum\limits_{k = 1}^{k} {N_{k} (x_{i} )_{d} } - k*x_{id} } \right| > > 0} \hfill & {if} \hfill & {x_{i} \in outlier} \hfill \\ \end{array}$$

Combining Eq. (2) with Eq. (3):

4 $$\begin{array}{*{20}l} {fluctuation(x_{i} ) \approx \sum\limits_{d = 1}^{D} {\frac{{x_{id} }}{{x_{id} + k \times x_{id} }}} { = }\frac{1}{1 + k}} \hfill & { \, if} \hfill & {x_{i} \in \;\;normal\;\; \, object} \hfill \\ {fluctuation(x_{i} ) = \sum\limits_{d = 1}^{D} {\frac{{x_{id} }}{{x_{id} + \sum\limits_{k = 1}^{K} {N_{k} (x_{i} )} }}} { < < }\frac{1}{1 + k}} \hfill & { \, if} \hfill & {x_{i} \in \;\;outlier \, \;\;and \, \;\; \, \sum\limits_{k = 1}^{K} {N_{k} (x_{i} )_{d} > > k \times x_{id} } } \hfill \\ {fluctuation(x_{i} ) = \sum\limits_{d = 1}^{D} {\frac{{x_{id} }}{{x_{id} + \sum\limits_{k = 1}^{K} {N_{k} (x_{i} )} }}} { > > }\frac{1}{1 + k}} \hfill & { \, if} \hfill & { \, x_{i} \in \;\;outlier \, \;\; \, and \, \;\;\sum\limits_{k = 1}^{K} {N_{k} (x_{i} )_{d} < < k \times x_{id} } } \hfill \\ \end{array}$$

If ***x***_***i***_ belongs to a normal object, its fluctuation value is approximately equal to 1/(1 + *k*); if ***x***_***i***_ belongs to an outlier, the fluctuation value will be significantly different from that of the normal objects.

**Figure 6:**
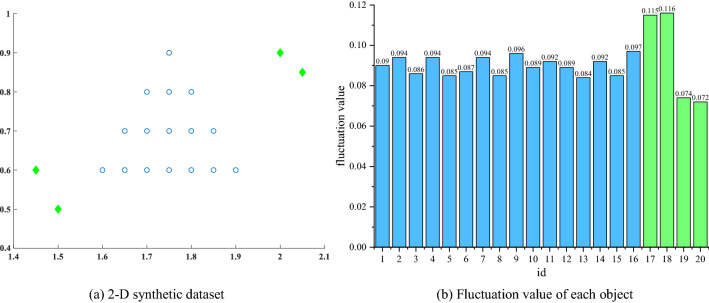
2D synthetic dataset and fluctuation value of each object. Sixteen normal objects are represented by blue hollow circles, and four outlier points are represented by solid green diamonds.

We use a two-dimensional synthetic dataset in Fig. 6 to demonstrate significant differences in the fluctuations of outliers and normal objects:

After the objects in the 2D synthetic dataset are propagated by feature values, their fluctuation values are significantly different due to two different generation mechanisms of outliers and normal objects. The fluctuation values of the two outliers located in the upper right corner are significantly higher than those of the normal objects, while the fluctuation values of the outliers located in the lower left corner are significantly lower than those of the normal objects. Based on the difference in fluctuation values, the outlier factor of the objects can be further calculated in the downstream task.

### Outlier factor

The outlier factor of ***x***_***i***_ is defined as:

5 $$OF(x_{i} ){ = }\sum\limits_{t = 1}^{T} { \, \sum\limits_{{x_{j} \in N_{k} (x_{i} )}} {\left| {fluctuation(x_{i} ) - fluctuation(x_{j} )} \right|} }$$

In Eq. (5), *T* denotes the number of generated graphs. Since a random connection is used in the graph generation process, multiple graphs are used to ensure the stability of the FBOD. The outlier factor of object ***x***_***i***_ captures the degree to which we call ***x***_***i***_ an outlier. The larger the OF value is, the more likely it is that the object is an outlier; the smaller the OF value is, the more likely it is that the object is normal.
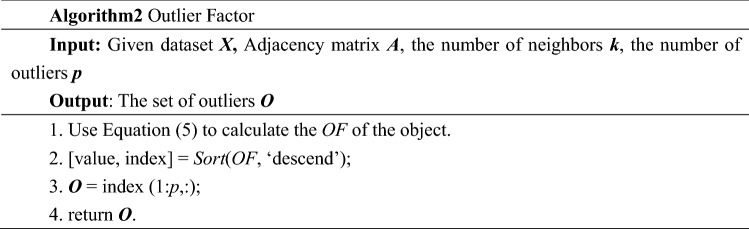


### Time complexity analysis

The steps of FBOD to detect outliers contain a total of 3 parts: graph generation, feature value propagation and fluctuations, and calculation of outlier factors. We analyze the time complexity of each part in detail:

- *Graph generation* The time complexity of randomly selecting *k* neighbors for *n* objects in the dataset is *O*(*k* * *n*).
- *Feature value propagation* The time complexity of each object in ***X*** aggregating the feature values of its neighbors is *O*(*k* * *n*).
- *Fluctuation* The original feature values of each object are compared with the feature values after feature value propagation with a time complexity of *O*(*n*).
- *Outlier factor* The time complexity of comparing the fluctuating values of each object with the feature values of its neighbors is *O*(*k* * *n*).

Since *k* is a constant, the overall time complexity of the FBOD algorithm is *O*(*n*).

## Experiments

To verify the effectiveness of the FBOD method, we compare the FBOD with several state-of-art algorithms in real datasets. The source code of our model is implemented in MATLAB 2019A. The hardware environment for the experiments is an Intel(R) Core(TM) i7-7700 3.60 GHz CPU with 16 GB of RAM. The operating system environment is Microsoft Windows 10 Professional.

### The summary of datasets and compared algorithms

ODDS (http://odds.cs.stonybrook.edu/) openly provides access to the collection of outlier detection datasets with ground truths in various real-world fields. We use the multidimensional point datasets in the ODDS and remove duplicate objects. All the datasets are summarized in Table 1. The following eight datasets are widely used in the literature related to outlier detection.

**Table 1:** Dataset statistics.

Dataset	# of records	# of features	# of outliers	Outlier ratio (%)
breastw	683	9	239	34.9
wbc	377	30	20	5.3
wine	129	13	10	1.2
heart	267	44	55	20.6
vowels	1452	12	46	3.1
lympho	148	18	6	4.1
pima	768	8	268	34.8
glass	213	9	9	4.2

We selected five different types of eight state-of-the-art outlier detection algorithms in for comparison experiments with the proposed FBOD. These algorithms in Table 2 are common types in the outlier detection field, and are used as comparison algorithms in most related literature. To compare the performance of each algorithm fairly, all algorithms are implemented in MATLAB 2019A.

**Table 2:** Comparison algorithm statistics.

Type of algorithm	Acronym of algorithm
Neuron network-based	AE, SO-GAAL
Graph-based	CutPC
Local outlier factor-based	LOF, COF
Clustering-based	K-means, OPTICS
Isolation-based	IForest

Due to the large variety and number of algorithms in the comparison experiments, the parameter settings for each type of algorithm are different. Therefore, Table 3 is used to describe in detail the parameter settings of each algorithm in the experiments.

**Table 3:** Parameter setting.

Algorithms	*k*(number of nearest neighbors)	Number of graphs	Learning rate	Number of iterations	Number of layers	*xi*(relative decrease in density)	minpts(Number of points required to form a cluster)	Number of isolation trees & subsample size
FBOD	2–100	1–5	\	\	\	\	\	\
AE	\		0.0001–0.002	10–100	3	\	\	\
CutPC	\	\	\	\	\	\	\	\
LOF	2–100	\	\	\	\	\	\	\
COF	2–100	\	\	\	\	\	\	\
K-means	2–100	\	\	\	\	\	\	\
OPTICS	\	\	\	\	\	0.1	2 –100	\
IForest	\	\	\	\	\	\	\	100, 56–256
SO-GAAL	\	\	0.0001–0.002	10–100	3	\	\	\

### Evaluation techniques

In real-world applications, ground truth outliers are generally rare. The receiver operating characteristic (ROC) curve, which captures the trade-off between sensitivity and specificity, has been adopted by a large proportion of studies in this area. The area under the curve (AUC), which ranges from 0 to 1, characterizes the area under the ROC curve. An algorithm with a large AUC value is preferred^35^. We choose the execution time, AUC, accuracy (ACC), detection rate (DR), and false alarm rate (FAR) as the algorithm performance evaluation metrics. Higher AUC, ACC, and DR values and lower FAR and execution time indicate better performance.

Let TP be the number of outliers correctly marked as outliers by the algorithm (TOP-*p*); TN be the number of normal objects correctly marked as normal by the algorithm; FP be the number of normal objects incorrectly marked as outliers by the algorithm; FN be the number of outliers that the algorithm incorrectly marks as normal objects. The calculation method of each evaluation techniques is shown in Algorithm 3.
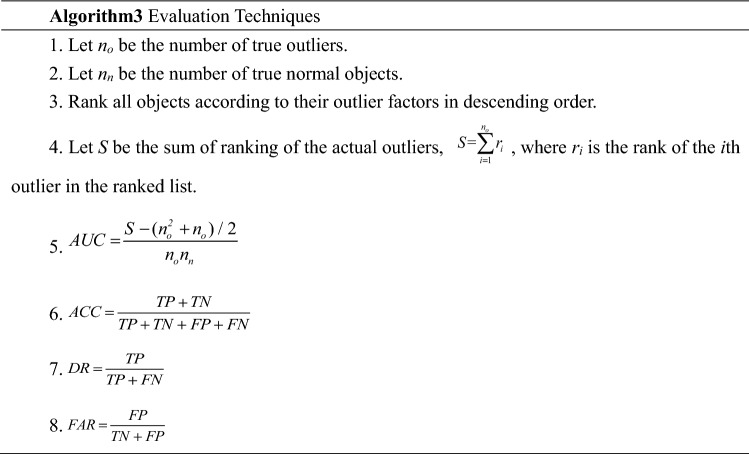


### Experimental on real-world tabular datasets

**Figure 7:**
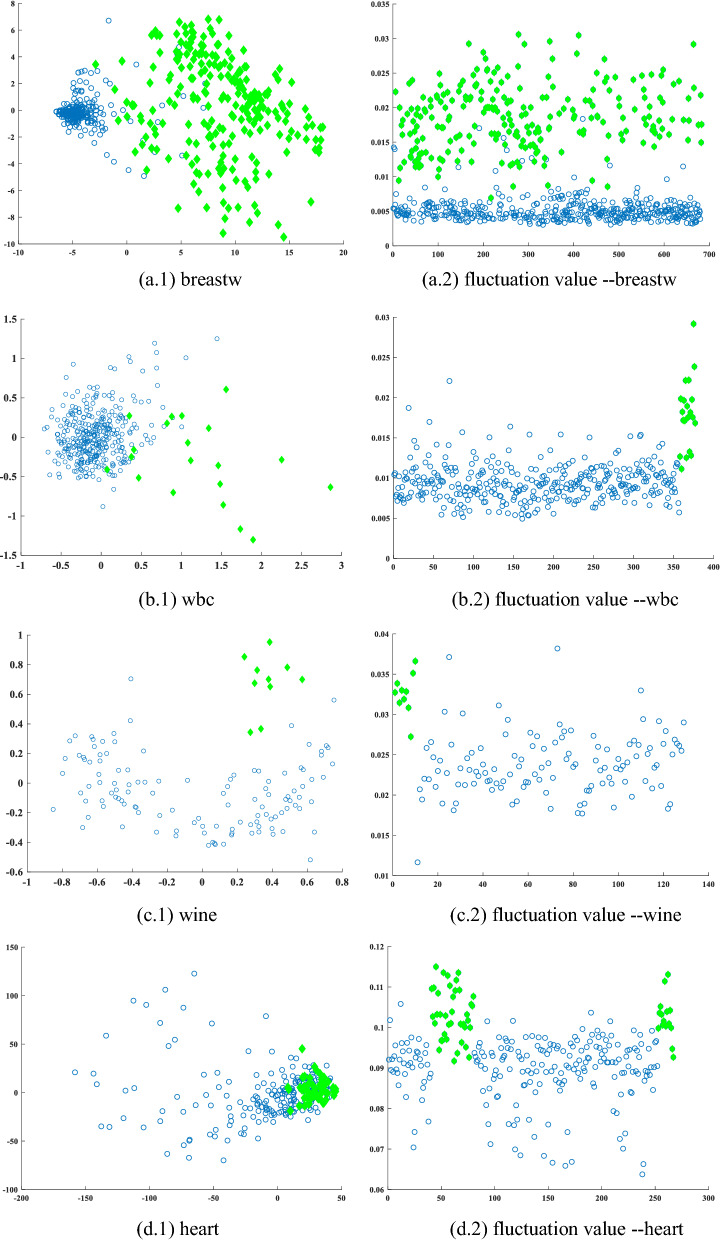

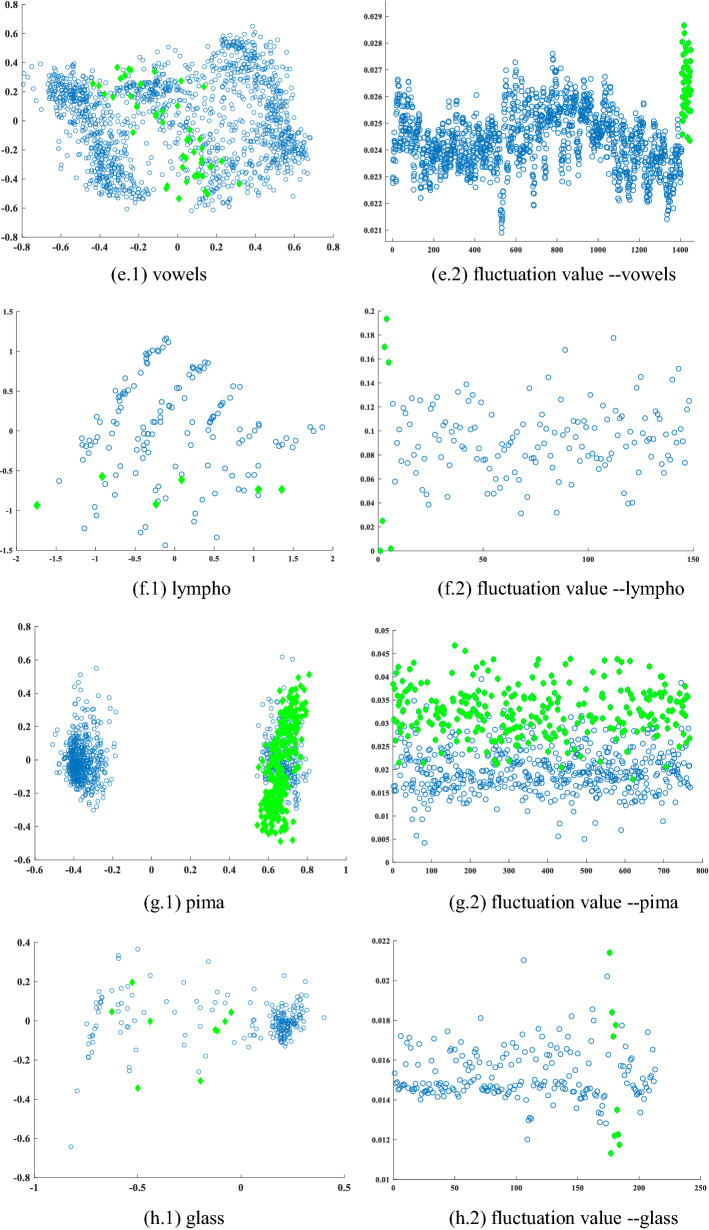
The original data distribution after PCA dimensionality reduction. The blue hollow circles represent normal objects, the green solid diamonds represent the true outliers in each dataset (left) and fluctuation values of each object in datasets, and the green solid diamonds represent the fluctuation values of true outliers in each dataset (right).

We judged the *p* objects (the size of *p* is equal to the number of true outliers in the dataset) with the highest outlier factor determined by the FBOD as outliers and compared them with the labels. Figure 7 shows the fluctuation values of each object as determined by FBOD. The left side of Fig. 7 show the original data distribution after dimensionality reduction using principal component analysis (PCA), and the right side shows the fluctuation values of each object determined by FBOD.

Figure 7 shows the breastw, wbc, wine, heart, vowels, lympho, pima, and glass datasets from top to bottom. There are many outliers in the original dataset that is mixed in the normal object area on the left. These outliers are usually difficult to detect by distance-based, density-based and clustering-based algorithms. On the right side of Fig. 7, after the feature value propagation, the fluctuation values of normal objects and outliers are significantly different; therefore, true outliers can be detected in downstream tasks based on the difference in fluctuation values between objects.

For each algorithm on each dataset, we adjusted the parameters and executed 20 experiments. In order to eliminate the influence of random factors on the experimental results, we repeated the experiment 30 times under the parameters corresponding to the best detection results obtained by the algorithm and used the average value as the final performance evaluation of the algorithm.

The experimental results of our proposed method and eight comparison algorithms on eight datasets are shown in Table 4. At the same time, we use the Table 5 to describe the parameters corresponding to the best detection results obtained by each algorithm.

**Table 4:** Experimental results on real-world datasets.

Datasets	(a) Actual execution time								
	Time (s)								
	FBOD	AE	CutPC	LOF	COF	K-means	OPTICS	IForest	SO-GAAL
breastw	**0.0026**	0.2353	0.1232	0.1539	1.2333	0.1240	**0.1215**	0.1692	0.168
wbc	**0.0028**	0.1404	**0.0370**	0.2016	0.5517	0.0824	0.0464	0.1372	0.193
wine	**0.0004**	0.0421	0.0325	0.0573	0.1698	**0.0062**	0.0096	0.1421	0.091
heart	**0.0023**	0.0990	0.0375	0.0798	0.3482	**0.0200**	0.0250	0.2282	0.078
vowels	**0.0107**	0.3380	0.1847	0.4356	25.981	**0.1272**	0.1757	0.1859	0.447
lympho	**0.0004**	0.0473	0.3153	0.1057	0.5457	0.2353	**0.0113**	0.1566	0.176
pima	**0.0030**	0.1944	0.0776	0.2131	23.531	**0.0360**	0.0594	0.1722	0.195
glass	**0.0006**	0.0626	0.0162	0.0820	2.3947	**0.0076**	0.0138	0.1285	0.045

Datasets	(b) AUC performance								
	AUC								
	FBOD	AE	CutPC	LOF	COF	K-means	OPTICS	IForest	SO-GAAL
breastw	**0.9906**	0.8911	0.9238	0.8131	0.6962	0.8814	0.8522	**0.9412**	0.6212
wbc	**0.9802**	0.9005	0.9529	0.9555	0.9328	0.9372	0.9405	**0.9705**	0.8533
wine	**0.9677**	**0.9437**	0.5286	0.9244	0.7790	0.7790	0.8542	0.9222	0.6791
heart	**0.5201**	**0.4758**	0.1910	0.2420	0.2314	0.3652	0.3321	0.2533	0.2032
vowels	**0.9186**	0.8102	**0.9889**	0.8480	0.8180	0.5554	0.9078	0.6829	0.5943
lympho	**0.9035**	0.5213	**0.9812**	0.7899	0.7840	0.8847	0.8635	0.8742	0.6892
pima	0.7823	**0.9322**	0.7798	0.4900	0.7363	**0.9510**	0.8893	0.6705	0.6697
glass	0.7719	0.5487	**0.8399**	0.7941	**0.8769**	0.4733	0.7216	0.7213	0.5543

Datasets	(c) Accuracy performance (%)								
	ACC(%)								
	FBOD	AE	CutPC	LOF	COF	K-means	OPTICS	IForest	SO-GAAL
breastw	**97.07**	94.33	94.43	68.66	91.50	68.66	68.66	**96.09**	68.66
wbc	**96.81**	94.51	95.22	**95.75**	94.69	94.69	95.22	95.40	94.69
wine	**94.83**	**91.73**	84.49	90.69	84.49	87.59	89.14	**91.73**	87.59
heart	**67.04**	61.29	59.55	61.04	61.04	**65.54**	64.04	59.80	61.04
vowels	**96.32**	94.21	**98.07**	95.73	95.86	94.07	95.86	94.62	94.35
lympho	**96.39**	93.69	**97.29**	94.59	94.59	94.59	94.59	94.14	93.24
pima	73.43	**84.63**	70.05	70.05	62.50	**95.83**	82.29	72.22	70.05
glass	**94.36**	92.17	92.48	92.48	**93.45**	92.48	92.48	92.80	92.48

Datasets	(d) Detection rate performance (%)								
	DR(%)								
	FBOD	AE	CutPC	LOF	COF	K-means	OPTICS	IForest	SO-GAAL
breastw	**95.81**	91.91	92.05	55.23	87.86	55.23	55.23	**94.42**	55.23
wbc	**70**	48.33	55.00	**60**	50	50	55.00	56.66	50
wine	**66.67**	**46.67**	0	40	0	20.0	30	**46.67**	20
heart	**20**	6.06	1.81	5.45	5.45	**16.36**	12.72	2.42	5.45
vowels	**42.03**	8.69	**69.56**	32.60	34.78	6.52	34.78	15.21	10.86

Datasets	DR(%)								
	FBOD	AE	CutPC	LOF	COF	K-means	OPTICS	IForest	SO-GAAL
lympho	**55.56**	22.21	**66.67**	33.33	33.33	33.33	33.33	27.78	16.67
pima	61.94	**77.98**	57.08	57.08	45.79	**94.02**	74.62	60.19	57.08
glass	**33.33**	7.41	11.11	11.11	**22.22**	11.1	11.11	14.81	11.11

Datasets	(e) False alarm rate (%)								
	FAR(%)								
	FBOD	AE	CutPC	LOF	COF	K-means	OPTICS	IForest	SO-GAAL
breastw	**2.25**	4.35	4.27	24.09	6.53	24.09	24.09	**3.00**	24.09
wbc	**1.68**	2.89	2.52	**2.24**	2.80	2.80	2.52	2.42	2.80
wine	**2.79**	**4.48**	8.40	5.04	8.40	6.72	5.88	**4.48**	6.72
heart	**20.75**	24.37	25.47	24.52	24.52	**21.69**	22.64	25.31	24.52
vowels	**1.89**	2.98	**0.99**	2.20	2.13	3.05	2.13	2.77	2.91
lympho	**1.88**	3.28	**1.40**	2.81	2.81	2.81	2.81	3.05	3.52
pima	20.40	**11.80**	23.00	23.00	30.00	**3.20**	13.60	21.33	23.00
glass	**2.94**	4.08	3.92	3.92	**3.41**	3.92	3.92	3.75	3.92

Values marked in bold are ranked by the top 2 in the dataset.

**Table 5:** Optimal parameter setting.

Algorithms	*k *(number of nearest neighbors)	Number of graphs	Learning rate	Number of iterations	Number of layers	*xi* (relative decrease in density)	minpts (number of points required to form a cluster)	Number of isolation trees & subsample size
FBOD	60	4	\	\	\	\	\	\
AE	\	\	0.0008	100	3	\	\	\
CutPC	\	\	\	\	\	\	\	\
LOF	50	\	\	\	\	\	\	\
COF	50	\	\	\	\	\	\	\
K-means	10	\	\	\	\	\	\	\
OPTICS	\	\	\	\	\	0.1	30	\
IForest	\	\	\	\	\	\	\	256
SO-GAAL	\	\	0.0006	70	3	\	\	\

In the eight real-world datasets, we compared the execution times of the eight algorithms. Observing Table 4, we can conclude that the execution time of the FBOD is much lower than that of the comparison algorithms. The average execution time of FBOD is only 5% of the average execution time compared to the OPTICS algorithm, which has the fastest average execution time. Furthermore, FBOD accounts for only four ten-thousandths of its execution time compared to the COF algorithm, which has the slowest average execution time.

The main reasons for the extremely low time overhead of FBOD are that (1) it does not require the calculation of distances or densities, and (2) it only calculates the difference between the fluctuations of the object itself and the fluctuations of its neighbors and does not involve the calculation of relationships with all objects in the dataset.

From Table 4b–e, several observations can be obtained:

1. FBOD obtains the best results in four datasets: breastw, wbc, wine, and heart. Since the heart dataset has 44 dimensions, the curse of dimensionality caused a near failure of the distance- or density-based methods.
2. The FBOD algorithm did not achieve the best detection results on the four datasets of vowels, lympho, pima, and glass. Combined with Fig. 7, it can be seen that the deviation of normal objects from outliers in these four datasets is small. Most of the outliers are mixed with the normal objects, and the resulting difference between the fluctuation values of the normal objects and the fluctuation values of the outliers is small, which affects the detection performance of the FBOD algorithm.
3. The average AUC value of FBOD on eight real datasets is 0.85, which is 6% higher than the next highest OPTICS method. The average AUC value of FBOD is 10% higher than that of the isolated forest algorithm. The effectiveness of FBOD is demonstrated by the excellent detection results achieved in different types of outlier detection tasks.

### Experiments on video data

The UCSD outlier detection dataset (http://www.svcl.ucsd.edu/projects/anomaly/dataset.htm) was acquired with a stationary camera mounted at an elevation, overlooking pedestrian walkways. In the normal setting, the video contains only pedestrians. Outliers are due to either:

- The circulation of nonpedestrian entities in walkways.
- Anomalous pedestrian motion patterns.

**Figure 8:**
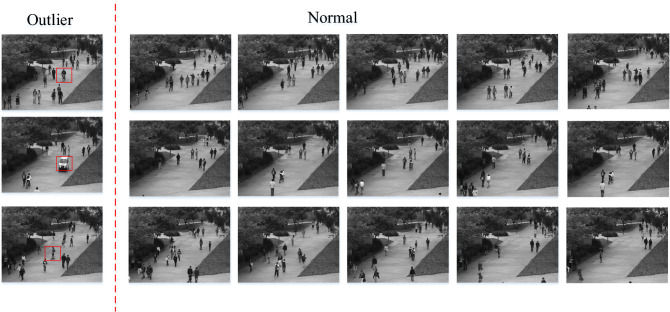
Video outlier detection dataset. The outliers on the leftmost are anomalous events on the sidewalk with bikers, small carts and skaters, and we have highlighted the cause of the anomaly with a red rectangle.

Commonly occurring anomalies includes bikers, skaters and small carts. All outliers are naturally occurring, i.e., they were not staged for the purposes of assembling the dataset. We constructed 3 datasets based on the UCSD library, and each dataset contained 60 normal images and 3 outliers. Finally, each dataset is transformed into a 37,604*63 matrix ***X***. Each column of ***X*** represents an object (a picture), and each row corresponds to the value of a pixel point at a certain location. Figure 8 shows a selection of photos from the video dataset used.

Most outlier detection algorithms are difficult to execute due to the high dimensional of the video dataset. Therefore, we only choose the AE, SO-GAAL algorithm in section "The summary of datasets and compared algorithms" for comparison experiments with FBOD. Each algorithm is executed 30 times, and the average value are selected as the final performance evaluation of each algorithm. The FBOD algorithm has features that make it possible to achieve effective detection in arbitrarily high dimensions. FBOD selects those images with the largest differences in pixel values as outliers based on the differences in pixel value between images.

**Figure 9:**
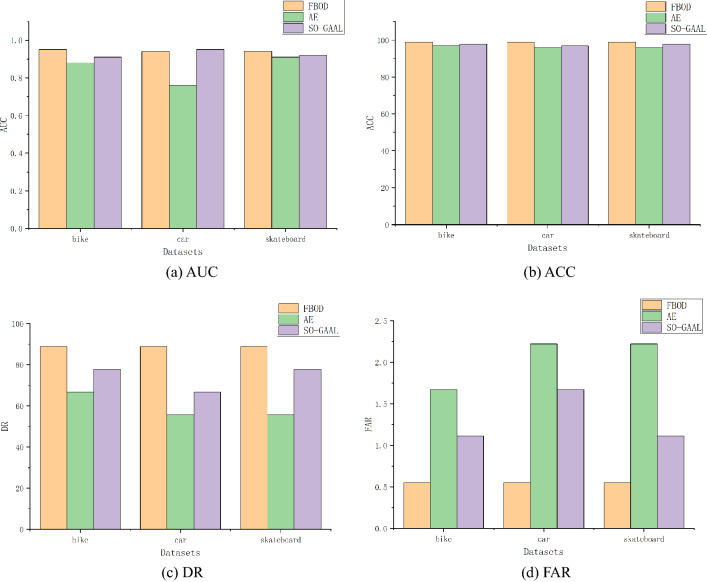
Experimental results of the three algorithms on video dataset.

**Figure 10:**
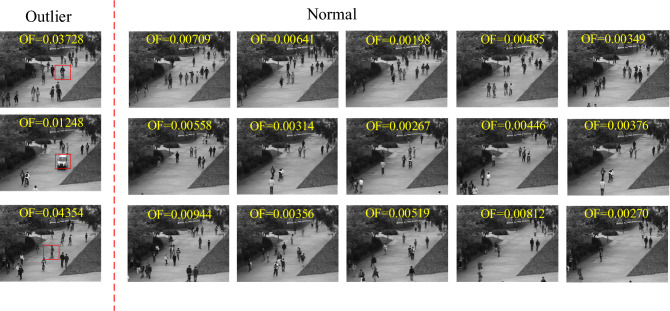
Visualization of FBOD detection results. The OF value of the leftmost outlier is significantly higher than that of the normal object.

Figure 9 shows the experimental results of the three algorithms. FBOD was able to detect the majority of outliers in the dataset. This is due to the large changes in pixel values of the images caused by anomalous behaviors such as bikers and small cars on the sidewalk. FBOD is able to detect outliers based on the different fluctuation values generated after aggregating neighbor information between images. Also, since the FBOD algorithm does not need to calculate distance and similarity between objects, that makes the FBOD have the potential to handle real-world video anomaly detection tasks. Figure 10 visualizes some of the detection results in the dataset.

### Research on the influence of parameters on the detection performance of FBOD

We conducted 20 experiments on each real-world dataset to investigate the effects of the number of neighbors *k* and the graph number *T* on the performance of the FBOD. The experimental results are shown below.

#### FBOD performance against various neighbor’s k

**Figure 11:**
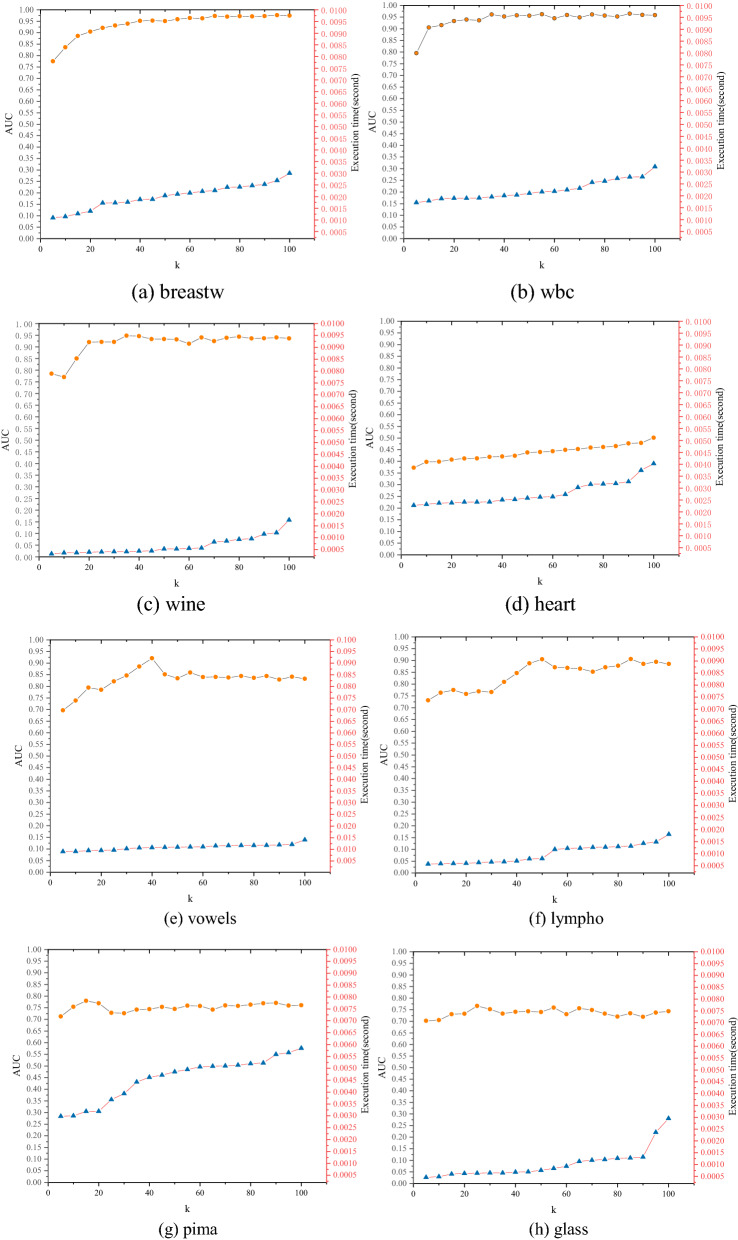
FBOD AUC performance (y1-axis) and execution time (y2-axis) versus the different neighbors’ *k* (x-axis).

Parameter *k* ranges from 5 to 100, and the value of *k* increases by 5 each time (graph number *T* = 2). Figure 11 shows that when *k* gradually increases, the AUC of FBOD also gradually increases. After reaching the highest AUC, *k* continues to increase, and the AUC value tends to stabilize. At the same time, when *k* gradually increases, the execution time of FBOD gradually increases.

#### FBOD performance against various graph numbers T

**Figure 12:**
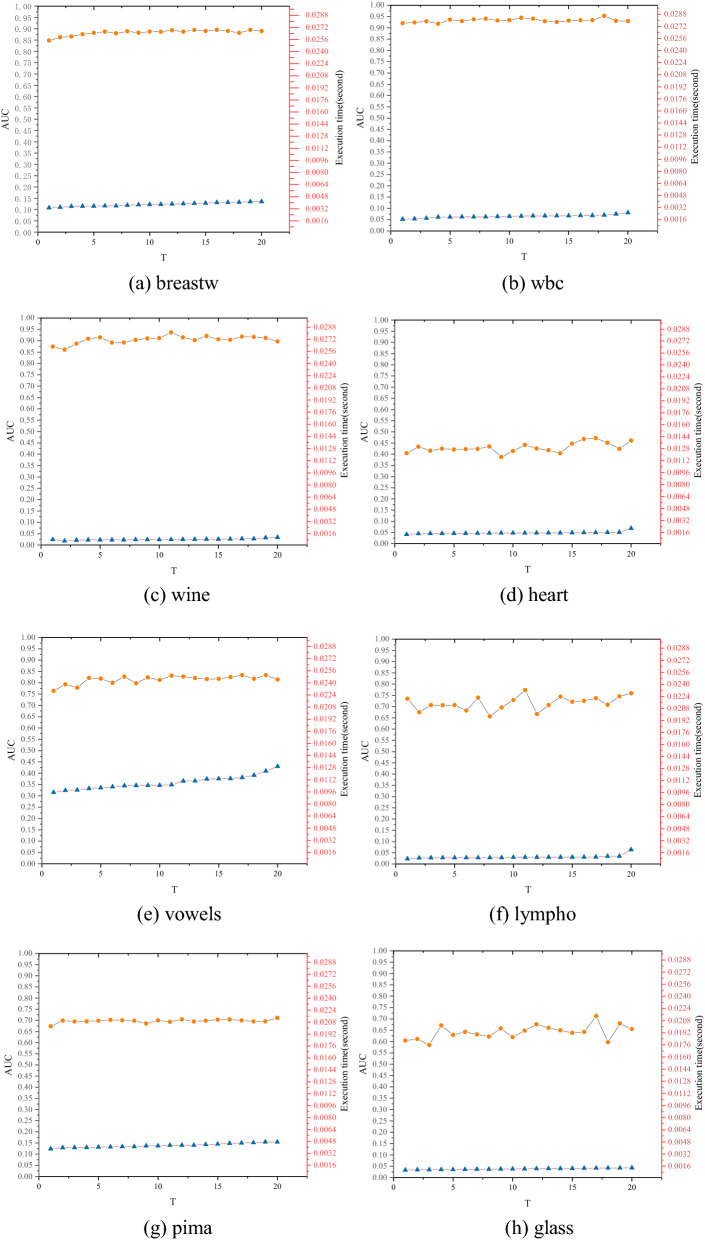
FBOD AUC performance (y1-axis) and execution time (y2-axis) versus the different graph numbers (x-axis).

Parameter *T* ranges from 1 to 20, and the value of *T* increases by 1 each time (*k* = 10). Figure 12 shows that when *T* gradually increases, the AUC of the four datasets of wine, heart, lympho, and glass are highly variable. The main reason for this phenomenon is that the distribution of normal objects in the dataset is too loose, which leads to a large variation in the selection of neighbors in each graph generation. Compared with the number of neighbors *k*, the parameter *T* has less impact on the performance of the FBOD; its main role is to fine-tune the performance and improve the robustness of the algorithm.

## Conclusion

In this paper, we proposed a fluctuation-based unsupervised outlier detection algorithm that changes the distribution of an original dataset by allowing objects to aggregate the feature values of their neighbors. Then, we define a new property of the object, fluctuation, in addition to distance, density and isolation. Finally, the fluctuation of the object is compared with its neighbors, and those objects with larger outlier factors are judged as outliers. FBOD is the first method that uses feature value propagation techniques and utilizes fluctuation for outlier detection. The results of experiments comparing FBOD with eight state-of-the-art algorithms on eight real-world tabular datasets show that FBOD achieves the best or next best AUC on six datasets. Meanwhile, FBOD achieves excellent detection results on video data. Most importantly, since the fluctuation-based algorithm does not need to calculate distance or density, the algorithm requires a very short execution time. It has high potential for real-worlds applications for outlier detection in large-scale data. Finally, we investigate the influence of hyperparameters in the FBOD on the detection effect in detail. However, the FBOD method still has some limitations, such as the need for the researcher's experience to manually adjust the hyperparameters. In the future, we will try to find the best settings for hyperparameters that can fit most practical applications as well as references for adjusting them. At the same time, we will attempt to take the FBOD algorithm by pretraining it to learn the fluctuation bounds of normal objects and then introduce it into the online application of data streams.

## Data Availability

The datasets generated and/or analyzed during the current study are available in the ODDS repository, http://odds.cs.stonybrook.edu/.
